# Recent Advances and Potential Applications of Atmospheric Pressure Cold Plasma Technology for Sustainable Food Processing

**DOI:** 10.3390/foods11131833

**Published:** 2022-06-22

**Authors:** Ximena Yepez, Alba E. Illera, Haci Baykara, Kevin Keener

**Affiliations:** 1 Escuela Superior Politécnica del Litoral, ESPOL, Facultad de Ingeniería Mecánica y Ciencias de la Producción, Campus Gustavo Galindo Km 30.5 Vía Perimetral, P.O. Box 09-01-5863, Guayaquil 090902, Ecuador; hbaykara@espol.edu.ec; 2 Faculty of Science, University of Burgos, Plaza Misael Bañuelos s/n, 09001 Burgos, Spain; aeillera@ubu.es; 3 Escuela Superior Politécnica del Litoral, ESPOL, Center of Nanotechnology Research and Development (CIDNA), Campus Gustavo Galindo, Km 30.5 Vía Perimetral, P.O. Box 09-01-5863, Guayaquil 090902, Ecuador; 4 College of Engineering and Physical Sciences, University of Guelph, Guelph, ON N1G 2W1, Canada; kkeener@uoguelph.ca

**Keywords:** cold plasma, sustainable technology, decontamination, non-thermal food processing, food safety

## Abstract

In a circular economy, products, waste, and resources are kept in the system as long as possible. This review aims to highlight the importance of cold plasma technology as an alternative solution to some challenges in the food chain, such as the extensive energy demand and the hazardous chemicals used. Atmospheric cold plasma can provide a rich source of reactive gas species such as radicals, excited neutrals, ions, free electrons, and UV light that can be efficiently used for sterilization and decontamination, degrading toxins, and pesticides. Atmospheric cold plasma can also improve the utilization of materials in agriculture and food processing, as well as convert waste into resources. The use of atmospheric cold plasma technology is not without challenges. The wide range of reactive gas species leads to many questions about their safety, active life, and environmental impact. Additionally, the associated regulatory approval process requires significant data demonstrating its efficacy. Cold plasma generation requires a specific reliable system, process control monitoring, scalability, and worker safety protections.

## 1. Introduction

The need to feed a growing population has changed the natural cycle of food, with the collateral consequence of generating enormous amounts of waste that gradually pollutes our world. The waste per capita in the world fluctuates between 0.1 to 4.5 kg per day, and the waste from food and greens reaches up to 44% of global waste [1]. The actual structure of food production requires an urgent change toward protecting our environment, and it can be achieved by keeping food, materials, and resources in the system as long as possible. This concept is not only focused on environmentally friendly production, but it also intends to add value to products and materials by diversifying their use [2]. Food production covers raw materials, processing, packaging, distribution, and waste management, and it has been a challenge to integrate each step of food production with this common goal.

**Figure 1:**
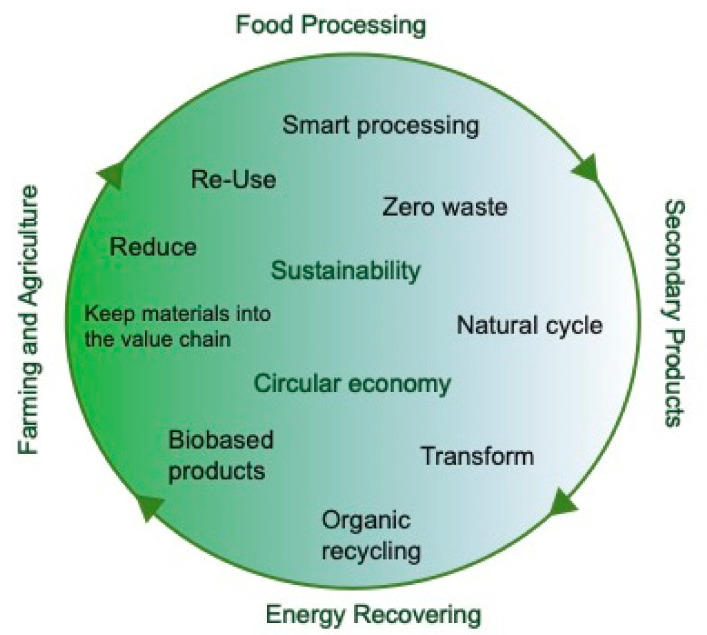
The role of cold plasma technology in a circular economy.

Non-conventional technologies such as Atmospheric Cold Plasma (ACP) play an essential role within the circular economy concept as it has the advantage of using renewable resources to produce safe and sustainable food products (Figure 1) [3]. The inputs in ACP processing technology are electricity and non-toxic gases. The ACP state can be formed by exposing gas to an electric field or electromagnetic waves that create a partial gas ionization (less than 2% ionized gas), which leads to the formation of reactive plasma species at room temperature. ACP can be generated from common gases such as air, oxygen, nitrogen, carbon dioxide, or other gases depending on the application with low energy input since the bulk gas is not principally heated. The reactive plasma species interact with biological surfaces, and, after the source of energy is disconnected, the reactive species come back to their original energetic state. Therefore, ACP is an environmentally friendly and non-thermal technology that does not requires consumables such as water or chemical additives.

This review aims to highlight the importance of cold plasma technology as an alternative solution to some challenges in the food chain, such as the extensive energy demand and chemicals the hazardous chemicals used. Specifically, it collects and analyzes the literature that relates the applications of cold plasma technology within a sustainable agriculture and food processing perspective, including (a) product cleansing and decontamination, (b) sustainable production improvement, and (c) potential risks and regulations.

## 2. Product Cleansing and Decontamination

According to the Food and Agriculture Organization (FAO), food waste from cereals constitutes 30%, for root crops, fruits, and vegetables, food waste reaches up to 40%, oilseeds, meat, and dairy are responsible for up to 20% of food waste, and for the case of fish, food waste reaches 35% of their total production [4]. Most of the food waste is due to postharvest losses and poor-quality standards. ACP has been proposed as a processing aid for microbial control, maintaining nutrients undamaged [5]. Design processes that utilize environmentally friendly processing technologies for decontamination and extend shelf life may reduce food waste. ACP technology has been studied mainly as a processing aid for biological systems for effective microbial decontamination, toxin removal, and pesticide degradation as shown in Table 1 [6,7].

### 2.1. Microbial Decontamination

Microbial decontamination in the food industry has usually been achieved through high-temperature treatments, yet these can carry the loss of food quality [21]. Therefore, research on non-thermal technologies for this purpose is increasing. ACP has been widely applied to different food and has shown its effectiveness in reducing common foodborne related pathogenic bacteria:

***Salmonella* spp.:** Infections caused by *Salmonella* spp. are a significant public health issue worldwide, both in industrialized and developing countries. *Salmonella* spp. is one of the most common isolated foodborne pathogens. It can be present in many different food products, but the primary source for *Salmonella* spp. infections are animals and their derived products, mainly poultry, eggs, and dairy products [22,23]. The infection manifestation caused by these bacteria can differ from common salmonellosis to bacteremia or typhoid fever [24]. Due to the impact of these bacteria on human health, extensive research on its inactivation in food has been recently carried out. For example, raw wheat was treated with ACP at 44 kV for 20 min, where a 4.8 log CFU/g reduction for a *Salmonella enterica* 5-strain mix was observed [9]. Lower inactivation results with maintained quality properties were found when treating tender coconut water at 120 kV for 120 s, achieving 1.3 log ± 0.3 reductions over *Salmonella enterica* serovar Typhimurium LT2 (ST2) 24 h after the treatment, where no product degradation was found [25]. The inactivation of bacteria was reflected by a morphological change in their structure after treatments, where the release of intracellular materials could be identified after scanning electron microscopy (SEM) analysis. Lettuce presents a high risk of *Salmonella* contamination, among other bacteria. Although no complete inactivation was achieved, a 4.5 log reduction of *Salmonella enterica* serovar Heidelberg was achieved after 10 min of exposure to the treatment of a two-dimensional array of DBD plasma [26]. The same treatment was applied on chicken breast, but bacteria reduction only reached 3.7 log. The difference was explained by the presence of a higher content of protein, which could consume plasma species, decreasing their effect on bacteria. Much research work is focused on poultry products due to their higher incidence of *Salmonella Typhimurium* [10,14]. Recent work was published reporting decontamination of duck eggs shell using ACP, where a 4.09 log cycle reduction was achieved in just 40 s of treatment at 12 kV [8]. Sudarsan and Keener reported the successful use of ACP with spinach, by the inactivation of *Salmonella enterica* serovars and *Escherichia coli* [27]

***Escherichia coli:****E. coli* is a broad group of bacteria where different strains can be identified according to their pathogenesis and the illness that they cause. The most severe infections are caused by the enterohemorrhagic *E. coli* group (EHEC) [8]. The main reservoirs of this pathogen are ruminants, particularly cattle. Therefore, *E. coli* can be easily found in fresh produce, such as green leafy vegetables, caused by cross-contamination during handling [27] or contact with contaminated water [13]. Green leafy vegetables are usually consumed raw. Therefore, nonthermal technologies are needed for effective decontamination while maintaining fresh product characteristics. A significant reduction of *E. coli* 0157:H7 was observed after a 5 min treatment with cold plasma treatment on kale leaves, remaining below the detection limit with no substantial change in color [28]. *E. coli* was also inoculated on red chicory leaves, and, after 30 min of treatment at 15 kV, a reduction of 1.35 MPN/cm^2^ was observed, being MPN the most probable number of colonies [29]. In another study, *E. coli* was inoculated on cherry tomatoes and strawberry surfaces [30], and then fresh produce was treated in-package by a plasma treatment at 70 kV. After just 1 min, a 3.1 log CFU/sample reduction was observed in the tomatoes and a 3.5 log CFU/sample reduction in the strawberries after 5 min. Coconut water was treated for 2 min at 90 kV, and after 24 h of refrigerated storage, *E. coli* was observed to have been reduced to 5 log CFU/mL when modified air was used. Inactivation of *E. coli* was explained by the interaction of formed species in gas plasma with the bacteria cell walls, where DNA and proteins were leaked, driving cell wall damage and inactivation [31]. After ozone treatment, a similar effect was observed on *E. coli* cell walls [32].

***Listeria monocytogenes*:***L. monocytogenes* can be present on a wide range of fresh produce due to contamination during the production and processing phase [33]. It can be found in a wide range of Ready-To-Eat products (RTE) [34]. Among *Listeria* spp., *L. monocytogenes* is the common one related to listeriosis [29]. A significant concern during the inactivation of *L. monocytogenes* of food products is their ability to survive at temperatures as low as 2 to 4 °C and their capacity to grow in low moisture or high salt content conditions [34,35]. Due to the prevalence of these bacteria in raw foods such as vegetable products or cured and deli meats, non-thermal treatments are the best alternative. Recently, ACP was applied to sliced dry-cured beef. After an initial inoculation of 5.71 log CFU/cm^2^, a 0.83 log CFU/cm^2^ reduction was found with a 5 min treatment at 25 kV [36]. Monitoring of *L. monocytogenes* biofilms in lettuce and cabbage was studied by Srey and collaborators. Cold oxygen plasma treatments were applied to these products and showed reductions from 5.9 log CFU/cm^2^ to 2.0 and 1.8 log CFU/cm^2^ for lettuce and cabbage, respectively. Moreover, no changes in color or texture were observed [37]. In a different study, tomatoes and strawberries inoculated with *L. monocytogenes* showed a total reduction of the bacteria on tomatoes and a reduction of 4.2 log_10_ CFU/sample on strawberries after a 2-min ACP treatment [27].

### 2.2. Toxin Removal

The presence of mycotoxins in food is a significant issue in food and agriculture production. Mycotoxins are low molecular weight molecules produced as secondary metabolites of some filamentous fungi [38]. Mycotoxins can contaminate different crops in the field and during storage and processing [39]. Mycotoxins of significant concern are usually produced by the fungus of the genera *Aspergillus*, *Penicillium*, and *Fusarium* [15]. Many of these mycotoxins have been carcinogenic, mutagenic, and genotoxic. The toxicity of each mycotoxin directly depends on its structure, and therefore the inactivation mechanism will be different [39]. In general, mycotoxins are unaffected by food processing operations, and high concentrations of these toxins have been found in the final product [17]. Historically, fungicides have been applied to control mold growth, but these are undesirable because of their environmental impact and potential chemical residues. Thus, there is a significant need for research on environmentally friendly technologies to reduce mold and toxin levels. Recent results of the effect of ACP on aflatoxins, fumonisins, and trichothecenes, some of the most harmful toxins, are shown herein:

**Aflatoxins:** Most ACP applications on mycotoxins are focused on aflatoxins (AF) since they are some of the most toxic and carcinogenic mycotoxins [39]. Aflatoxins are mainly produced by *Aspergillus* species and are usually present in crops, cereals, seeds, nuts, and spices. The most representative toxins of this group are AFB1, AFB2, AFG1, and AFG2. The toxicity of aflatoxins is related to their lactone ring and the double bond present in the difuran ring moiety [7], being AFB1 the most toxic [40]. The effectiveness of plasma technology on this toxin has been extensively demonstrated. After a 40 min ACP treatment on pure aflatoxin solution (AFB1), no presence of toxin was observed, and 50% of the analyte disappeared after 10 min at 6 kV using helium as gas [41]. In the same study, corn was treated for 10 min, and a 65% reduction of AFB1 presence was observed. In a different report, the effect of ACP was studied on aflatoxin production in groundnuts with artificially inoculated *Aspergillus* [42]. The results showed that the production of AFB1 was reduced by 90% after a 12 min treatment at 60 W. The authors explained that the survival capacity of spores depends on their protective coat, which can be attacked by oxygen radicals, causing aggressive oxidation and denaturalization. Similarly, raw peanuts were treated using an ACP jet system. After just 2 min and 5 min of treatment, a 23% and 38% reduction of AFB1 were achieved, respectively. Moreover, no significant changes in the product quality of peanuts samples were observed [15]. Shi and collaborators studied the effect of High Voltage Atmospheric Cold Plasma (HVACP) on aflatoxin present in corn. Totals of 62% and 82% of toxin degradation results were obtained after 1 and 10 min of HVACP treatment, respectively [43]. The proposed mechanism of degradation was related to the action of different reactive gas species on the C8-C9 bond of the furan ring, which was reported to be responsible for the aflatoxin toxicity [15].

**Fumonisins:** Among this group, different toxins can be found, where FB is the most common, specifically FB1 and FB2. Fumonisins are mainly produced by *Fusarium* species and are usually present in maize and its products [7]. The fumonisin structure is composed of a 22 carbon aminopentol with two tricarballylic acid side chains and a free amino group, which are responsible for fumonisin toxicity. Applying ACP on food products for fumonisins decontamination is less common, yet some examples can be found in the literature. For instance, in the study of Wielogorska and collaborators, after a 60 min immersion of 2 g of corn in a 200 µg/mL FB1 solution, the corn was treated for 10 min using an ACP jet system with helium as gas. A 64% reduction of the FB1 concentration in corn was reported after the treatment, and a total reduction was observed at the same time when a pure FB1 solution was treated, showing that the matrix where the toxin is present is an essential factor in its mitigation [17]. This was also demonstrated in another study, where different solutions of pure mycotoxins were treated, and total degradation of FB1 was reached in just 10 s after treatment with air plasma at 38 kV. The toxin structure was significant for the degradation of success [44]. In another study, date palm fruit was artificially inoculated to produce FB2 and treated by an argon double jet ACP. Total degradation of the toxin was achieved in 6 min [18].

**Trichothecenes:** Trichothecenes are a broad group composed of toxins such as deoxynivalenol (DON), nivalenol (NIV), or T-2 toxin. Trichothecenes are produced by many fungi species, such as *Fusarium*, *Trichothecium*, *Myrothecium*, or *Trichoderma* [45]. These toxins are usually found in crops, and their structure is characterized by a 12,13-epoxy-trychothec-9-ene nucleus, essential for their toxicity [46]. Their toxic effect is based on their ability to interfere with the synthesis of proteins, causing nausea, weight loss, and liver damage, among others [7]. Most of the existing studies on this group are focused on reducing DON. DON destruction in barley is a problem for malting-related industries. An ACP jet was used to produce plasma-activated water (PAW), and both raw and germinating barley were immersed from 5 to 20 min. No significant differences were observed based on immersion times, and reasonably high reduction rates were obtained after just 5 min in contact with PAW, where DON reduction results reached 22.5% and 34.6% for raw and germinating barley, respectively [47]. In a different study, PAW (10–40 min) was used for soaking the grain and mixing the dough for wheat sprouted bread preparation, and DON levels of the final product were maintained below the required standards, while bread quality was improved [48]. Abbasian and collaborators [49] demonstrated that argon plasma jet had an effective destruction effect on DON and *Fusarium*. DON inactivation was much faster when treated as a pure solution, achieving a total degradation after just 20 s of ACP treatment at 38 kV [44]. In some studies, DON has shown a higher resistance to destruction by plasma treatments. After a 60 min treatment at 6 kV on a pure DON solution, only 50% of the toxin was mitigated, compared to the total inactivation of other toxins in the same conditions and shorter treatment times, showing that the structure of the toxin is an essential factor for inactivation [17].

### 2.3. Pesticides Degradation

The presence of microorganisms, insects, or pathogens is essential for quality and economic loss in fresh products such as fruits and vegetables. The use of pesticides is the most common technique for reducing the incidence of these contaminants. When used improperly or intensively, it may lead to an excessive accumulation in the food product and the soil and water, becoming an environmental issue and a risk to human health [50]. Thus, there is an increasing potential for effective non-thermal technologies for removing these hazardous components while maintaining product quality and soil integrity. In this regard, ACP technology is starting to gain importance [51]. The research focused on pesticide degradation is scarce, especially when these are present in food [52]. ACP technology has been shown to degrade pesticides to safe or less toxic structures. Almost all the related studies attribute the degradation of pesticides to oxidation processes due to reactive species created in plasma, including H_2_O_2_, O_3_, O, H, and OH radicals [3]. The literature results are presented depending on the medium where the pesticide is present, differentiating between when they exist in a food matrix or a different matrix. As explained previously, matrix components can interfere with the ACP effect on the target product.

**Pesticides in water:** Many studies are focused on the degradation of pesticides in the aqueous medium due to the importance of these contaminants on wastewater. In 2016, Sarangapani and collaborators conducted a study where the degradation of three different pesticides, dichlorvos, malathion, and endosulfan, was tested. The degradation of these pesticides increased with higher voltage and longer treatment times, and the best results were obtained after 8 min at 80 kV, reaching degradation values of 79.0%, 69.6%, and 57.7%, respectively. GC/MS analysis determined that the intermediates created were less toxic than the original pesticides due to more specific chemical groups [52]. Moreover, alachlor was degraded in water using an 80 kV ACP treatment, producing total degradation after a 30 min treatment using dry oxygen. In their study, the authors determined that the decomposition of alachlor occurred due to the loss of aromaticity through a radical oxidation mechanism [53]. In a different study, where a non-thermal plasma needle with argon gas was employed for dimethoate removal from water, it was found that one of the decomposition products, omethoate, was more toxic than the original pesticide. Nevertheless, the overall toxicity in the water had decreased [54]. They also concluded that the addition of H_2_O_2_ to the solution increased the removal rate due to an increase in the reactive species formation. In the study carried out by Giardina and collaborators, two herbicides: mesotrione and metolachlor, were treated in water using DBD “pure air” plasma at 18 kV. The herbicide degradation showed to be dependent upon the treatment time and the initial concentration of the pesticide. A 20 min treatment was required for total degradation of mesotrione, and 45 min for metolachlor, while the necessary time was 30 min when treated together. Moreover, the authors compared pesticide degradation when using deionized water or tap water, which can have a closer composition to actual wastewater. Interestingly, no difference in herbicide degradation was found [55]. Although the degradation route for each pesticide will differ depending on their chemical composition and structure, the discussion carried out in the literature studies always points out the interaction of the formed reactive gas species with those compounds. After the plasma discharge, and depending on the plasma source used, plasma species will be formed in the gas phase and diffused into the liquid, or directly generated in the liquid. Once the reactive species are in the liquid phase, they will interact and, in most cases, degrade the pesticides. The exact degradation mechanism for each chemical compound would need to be studied by the analysis of structural changes, which is directly related to their toxicity. Although not many studies try to comprehend these mechanisms in depth, the study carried out by Wang et al. (2021), gave some interesting insights. They used ACP for the degradation of benazoxystrobin in water using different voltages and gases (air and oxygen). Best degradation results were found at 60 V after 9 min of treatment for both gases. An interesting result that this study provides is the difference in the pesticide degradation when the two different gases are used. When air was the gas, 60% of degradation was observed, while 90% degradation was reached when oxygen was used. The degradation of this compound was reflected in the destruction of the benzene ring structure, and the study results indicate a stronger effect of oxygen reactive species rather than nitrogen ones (main ones in air plasma) over this structure [56]. Similar inactivation mechanisms could take place when treating compounds with similar structures, but further studies in this field are needed for a better comprehension.

**Pesticides on food:** The studies focused on the degradation of pesticides present in food are commonly focused on fresh produce. The body of literature focused on the degradation of pesticides in food is smaller than that on the degradation of pesticides in water. The reason is the complexity of the matrix and the difficulty of measuring and interpreting results. In 2014, an in-package degradation of pesticides in strawberries was performed after a 15 s immersion in a solution containing a mix of pesticides: azoxystrobin, cyprodinil, fludioxonil, and pyriproxyfen. The strawberries were filled in a package with air, and DBD ACP was applied at 80 kV from 1 to 5 min. The highest reduction of pesticides was achieved after the 5 min treatment, which produced reductions of 69%, 45%, 71%, and 46%, respectively. The pesticide reduction was dependent on voltage and treatment time [51]. In a more recent study, the degradation of chlorpyrifos and cypermethrin sprayed on mango fruit was studied. The removal yield was 74% and 62.9% after 5 min of treatment. In this study, the plasma setup consisted of a gliding arc and argon micro-bubble distilled water [51]. The reduction of pesticide concentration is usually the main objective in these studies, but when the sample is a food product, quality properties must also be considered. In 2019, a DBD treatment was applied to tomatoes for chlorpyrifos degradation. The best results were observed after 6 min of treatment with a 5 W power, being pesticide reduction of 89.2%. Although the color index of tomatoes was enhanced, a depletion of carotenoids and phenolic content occurred [20]. In a different study, using plasma-activated water, pesticide removal and quality properties were investigated in grapes [57]. Other PAW treated times were tested, showing that the 30 min PAW performed best, with a 73.6% reduction. The treatment did not significantly affect grape properties such as vitamin C, sugar content, color, and firmness. In 2020, Cong and collaborators used a DBD cold plasma device for the degradation of malathion and chlorpyrifos in lettuce, obtaining a degradation yield of 53.1% and 51.4%, respectively [19]. Additionally, the authors analyzed the degradation pathways of both compounds. For this purpose, they analyzed the intermediates formed by the cold plasma treatment of the molecules such as the cleavage of P=S and C-S bonds and forming new compounds reacting with plasma activated species. The intermediates provide some information about the degradation mechanisms of the molecules, but further mechanistic studies could be carried out for a better understanding of cold plasma-aided degradation.

## 3. Sustainable Production Improvement

In addition to microbial control, ACP has been reported as a technology that can increase supply chain performance. Better utilization of materials is of great importance for the agriculture and food sectors.

### 3.1. Plasma Activated Liquids

When applied directly over the product to be treated, cold plasma reactive gas possesses certain limitations, including the half-life of many species and their limited contact with microorganisms and biofilms on surfaces. As an alternative, capturing plasma properties through the generation of plasma-activated liquids (PALs) is an emerging area of study extensively researched in the past few years. Recent publications on PALs have reported on a wide range of potential applications for their antimicrobial and agronomic properties. PALs chemistry can be tailored by adjusting the plasma generation characteristics of electricity, liquid, and gas composition.

ACP treatment can produce a wide variety of species depending on the environment in which cold plasma is applied. If this environment contains water, then one type of the essential reactive species formed is composed of hydroxyl radicals formed due to the electron impact ionization of water molecules. The radicals formed during the cold plasma treatment can react with other compounds such as hydrogen peroxide (H_2_O_2_), which is the product of the collision of hydroxyl radicals. Hydrogen peroxide can be formed through an electric field at low temperatures due to its thermal instability. Thus, if water is treated with ACP, the species mentioned above and H_2_O_2_ can be formed. These species have been detected in the liquid phase of plasma-activated water and have been determined to contain highly oxidizing ability in especially biological systems [58,59,60].

Liang et al. used Fourier infrared and optical emission spectroscopic techniques to determine reactive species such as hydrogen peroxide, NO_2_^−^, and NO_3_^−^ formed during the cold plasma treatment of different ratios of O_2_/N_2_ in a liquid environment. Results showed that oxygen-related species had increased when the O_2_ amount was higher than N_2_. The authors obtained the same results in the case of nitrogen, forming more nitrogen-based reactive species when the N_2_ ratio was higher [61]. Therefore, ACP applied to liquids can form different reactive species such as OH radicals, atomic oxygen, ozone (O_3_), NO_3_^−^, NO_2_^−^, N_2_O, NO, and NO_2_ depending on the atmosphere of the gas or gas mixtures used. These species have significant importance in cell proliferation, depending on the concentration. It has been demonstrated that a high level of reactive oxygen species can cause cell death and is effective against bacteria, fungi, cancer cells, and viruses [62,63].

**Figure 2:**
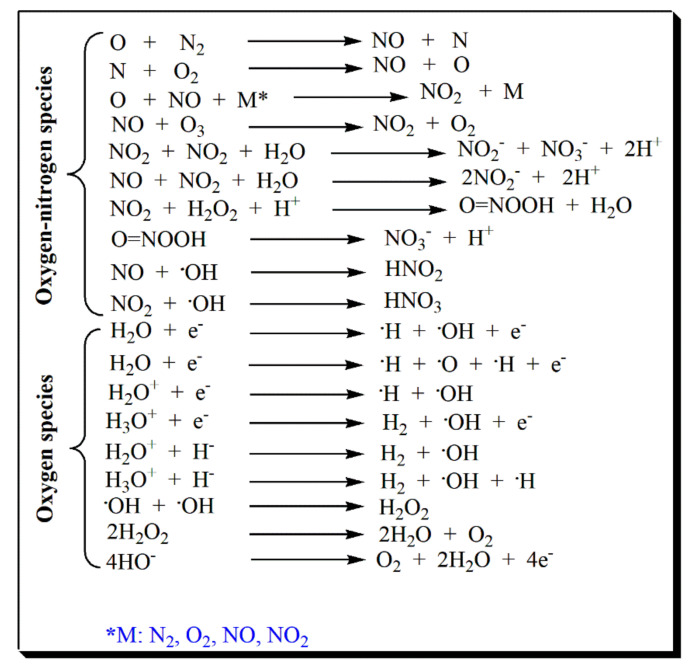
Potential reactive species formed with cold plasma treatment, using water and air atmosphere (N_2_ + O_2_).

ACP-treated liquids, especially water, become highly chemically reactive due to the species formed. Water and other plasma-activated liquids have a potentially reactive effect on living systems, such as media, hydrogen peroxide, saline, and organic acid solutions [64,65]. Consequently, plasma-activated liquids have drawn the attention of researchers because of their potential applications in medicine and biology. Lu et al. [66] treated deionized water with air spark and glow discharge cold plasma techniques and analyzed the resulting reactive species after treatment. Observations showed that each method produced different reactive species in the resulting deionized water. On one side, spark cold plasma formed H_2_O_2_ and NO_3_^−^, while on the other, glow discharge generated NO_2_^−^ and NO_3_^−^. This constitutes an interesting observation, as variations in conditions such as voltage/discharge can produce different reactive species in the same medium. As mentioned previously, the dose of the reactive species is critical for the proliferation or damage that such reactive species can cause in cells. It has been suggested that cold plasma discharge can control the type and perhaps the concentration of the reactive species produced. Moreover, the dosage of the reactive species resulting in ACP-treated deionized water can be controlled by mixing different discharges [66]. Tachibana and Nakamura [67], Lu et al. [66], and Tarabová et al. [68] explain that the formation of potential species in deionized water can occur by evaporation of water molecules through heat generated during the discharges and ion bombardment. Figure 2 summarizes the main reactive species reported in the literature [66,68].

### 3.2. Cold Plasma in Agriculture

Several applications of ACP in agriculture have been studied in seeds, plants, soil, and irrigation [69]. A fluidized bed of air in a DBD was tested in tomato seeds. Results showed that ACP treatment enhances the germination rate. For instance, a 5 min direct treatment of seeds obtained a root length of 28 mm compared to a 10 mm for the control [70]. The use of PAW to irrigate lentil seeds was able to increase root length by 128% in 6 days of growth in comparison with the control. Nitrates, nitrites, ammonium, and hydrogen peroxide were the chemical species measured in PAW and correlated to the enhanced growth [71]. In addition, it is suggested that modification and disruptions of the seed surface cause an increment in water uptake and permeability that may lead to better growth [72]. Similar results have been found in artichoke [73], pea [72], and soybean [74] seeds. Direct treatment of seeds and the use of PAW for irrigation are potential applications of ACP in agriculture. In 2022, a comprehensive review, Panka and collaborators reported about the use of cold plasma in sustainable seed production, and plant growth improvement [75]. While their study outlines some of the most relevant contributions in the field of cold plasma technology, it focuses heavily on the plant and seed aspects of the agricultural chain, and unfortunately does not provide major details on the latest developments of technologies in use for food processing applications, nor does it mention the use of plasma-activated liquids, a key application currently highly researched, in agriculture and decontamination.

Waste from food and greens makes up to 44% of global waste, followed by dry recyclables that include plastic, paper, and glass [1]. In a circular bioeconomy, waste is considered a source of nutrients that can be processed or extracted to obtain valued products. There is an opportunity to reduce food waste by processing food that has not met quality standards to obtain nutrients. Moreover, food losses from production, postharvest handling, storage, and processing stages, can be used to create new products.

The use of cold plasma to extract nutrients that provide physiological benefits has been studied recently. A compound with poor natural functional properties can be enhanced by applying ACP treatment, and this is the case with flavonoid naringin. An ACP treatment of 10 min with a dielectric barrier discharge system allowed to increase its radical scavenging capacity from 1.5% to 38.2%, which was correlated with a higher content of phenolic compounds [76]. ACP treatment also contributes to inhibiting tyrosinase with naringin treated with ACP for 10 min, from 6% to 83%. The ACP-treated naringin exhibit antioxidant, antimicrobial, and tyrosinase-inhibition properties. The extraction of materials with enhanced functional properties can be applied not only as food ingredients but also in cosmetics.

The use of biotechnology to produce biofuel has been studied as an essential source of renewable energy that potentially replaces fossil fuel. The goal of using algae for biodiesel production is to reduce carbon emissions at a competitive price. One of the significant challenges of biodiesel production from algae is the process yield. Almarashi and collaborators have studied the use of ACP treatment to increase lipid production yield and cellular growth of microalgae [77]. A short treatment time of 30 s for Chlorella Vulgaris using a plasma jet system with an energy consumption of 12 W increased cell viability by 26.6%. It is known that ACP treatment reduces the microbial load by membrane disruption, DNA damage, electroporation, or protein modulation [6]. However, a small dose of reactive plasma species may: (1) separate cellular clumps for a uniform distribution, (2) stimulate cellular defense mechanisms preventing death in the adaptation stage, or (3) enhance growth by receiving a low dose of nitrogen oxides [78]. Consequently, higher lipid content was produced by an ACP treatment of algae for biodiesel extraction.

### 3.3. Cold Plasma in Food Processing

The use of nitrites for processing cured meats is required for flavor and color development and microbial and rancidity control. The formation of the pink color in cured meats depends on the reactions of nitrites with the heme group of hemoglobin. However, a chain of reactions needs to occur in the first place, which includes the formation of nitric acid and then nitric oxide. The interaction of a reducing agent with nitric oxide can form nitrosyl myoglobin, which provides the pink color of cured meats. The use of ACP treatment has been studied as a technique to infuse nitrites in cured meats using nitrogen gas to develop a pink color without food additives. An ACP treatment of meat batter for 30 min increased nitrite to 66 mg/kg, developing the desired pink color [78]. It has been proposed that nitrogen plasma species such as nitrogen oxides NO_2_, N_2_O_3_, or N_2_O_5_ can form nitrites. It has been proposed that nitrogen plasma species such as nitrogen oxides NO_2_, N_2_O_3_, or N_2_O_5_ can form nitrites. Therefore, the nitrites formed during the APC treatment under ambient air atmosphere can cause the formation of nitrosyl hemochrome, which is responsible of the thermally treated meat pink color [79,80]. Furthermore, nitrimyoglobin was identified as a product of ACP treatment when a standard of hemoglobin was used to label the reaction of nitrites with the heme group. Nitrimyoglobin produces the green color in meats, yet it can be used in a higher concentration and as a reducing agent to form the pink color [80].

A study showed that ACP treatment increases the content of anthocyanin from 21% to 35% in pomegranate juice treated for 7 min with an argon gas plasma jet [81]. It is suggested that plasma reactive species may disrupt cell membranes that allow better extraction of micro components. The development of ACP to extract functional components is a promising application in food processing.

Modifications in protein structure with ACP have been reported with applications that include enzyme deactivation, dough rheological behavior modifications, surface functionalization in protein-based films, or a potential to reduce the immunoreactivity of specific proteins. The interaction of plasma reactive species with proteins showed changes in primary and secondary structures, cleavage, polymerization, aggregation, and oxidation [82]. Moreover, a recent review reported the use of reactive species from direct ACP treatment or PAL’s as a method to modify the structure of starch [83]. Currently, the main processes used to modify starches are the chemical methods, using harsh acids such as sulfuric or hydrochloric acid. Introducing the use of a non-conventional technology is interesting because it may reduce the use of chemicals in the food industry.

The structure of molecules can be modified by nitrogen or oxygen plasma species and by gases such as hydrogen. Soybean oil treated with a modified atmosphere of nitrogen–hydrogen gas was treated with high voltage ACP to increase the oil viscosity and change the fatty acid composition. The effect of ACP treatment was focused on the reduction of polyunsaturated fatty acids and increment of saturated fatty acids without the formation of the undesired trans isomers [84,85]. Changes in the chemical structure of vegetable oil are desired for applications such as frying and formulations that require provide specific oxidation stability or higher melting points in food products. ACP treatment can be used as a processing technology that can modify the chemical structure of vegetable oils.

## 4. Potential Risks and Regulations

Plasma reactive species are responsible for microbial decontamination and specific structure modifications, and they may facilitate nutrient extraction from biological systems. The atmospheric gas composition before and after an ACP treatment is similar, as the reactive species go back to their original gas state when the energy source is disconnected over a period of seconds, minutes, hours, and days, depending on the treatment conditions and type of product being treated.

Ozone generators are ACP devices. In an ozone generator, pure oxygen or dry air is exposed to an electric field leading to the formation of atomic oxygen, which stabilizes to form Ozone (O_3_). Ozone is classified as a Generally Recognized as Safe (GRAS) in the United States [86]. This means that it can be applied without restrictions or labeling. The final rule from the FDA providing GRAS approval in fruit and vegetable processing was given in 2001. The USDA final rule granting GRAS approval for ozone use in meat, poultry, and egg product manufacturing occurred in 2002 [86]. However, ozone is highly degrading of food quality and food manufacturing equipment. Thus, its commercial use has been limited in food processing.

Fortunately, not all ACP devices are solely ozone generators. Depending on the ACP device, the product being treated, the working gas (air vs. pure oxygen), and the exposure time, there is potential for a wide range of ionized gases and reactive species to be formed. In many cases, it is desirable to shift the ACP reactive species to alternatives other than ozone due to ozone having a high oxidation effect on food quality. As the ACP reactive species interact with the surface of the substrate, they may form measurable concentrations of common compounds such as peroxynitrites, nitrates, nitrites, or peroxides that remain in the environment or on the product for a few hours to allow continued residual fungicidal, bactericidal, virucidal (including COVID-19) activity significantly reducing the contamination levels without affecting the food quality, and in many cases, extending shelf-life. The wide range of potential effects resulting from the interaction of ACP device, product, working gas, and treatment necessitates experimental validation for each process currently to achieve Food and Drug Administration (FDA) regulatory approval. FDA is the regulatory approval agency for overall food safety review under the Federal Food, Drug, and Cosmetic Act (FD&C Act) for processed food article treatment. If one pursues a cold plasma treatment of raw agricultural products, the regulatory approval will fall to the Environmental Protection Agency (EPA) under the Federal Insecticide, Fungicide, and Rodenticide Act (FIFRA). Suppose the product being treated is a raw product that also is being graded, such as apples. In that case, Agricultural Marketing Service (USDA-AMS) must approve the process as the government agency tasked with quality inspection and cold plasma treatment may impact product quality. Interagency memorandums of understanding (MOUs) articulate the regulatory review procedures when a technology evaluation falls under multiple government agencies. There are standard processes of technology review and treatment classifications for each regulatory agency beyond this chapter’s scope; however, an example would provide useful details. If one considers the introduction of a cold plasma process to extend the shelf-life of strawberries from seven to 14 days. What would be the potential options for approval? If the cold plasma process is not making any claim on bacterial reduction, then it likely will not fall under EPA review. Thus, it would default to FDA safety review, which is relied upon by USDA-AMS in making their approval. Within the FDA regulatory review process, there exists an option called “Generally Recognized as Safe (GRAS) Self-Affirmation”. This is generally the preferred pathway of new technology adoption. In brief, this process allows a technology developer to design and finalize a cold plasma treatment for strawberries and once finalized collect relevant data on the residues, if any, existing on the treated strawberries and on the control strawberries. Additionally, nutritional information would be collected along with shelf-life data. Again, bearing in mind the intent of the process is to provide increased shelf-life without claim on any specific bacterial or pathogen reduction, one can assemble an expert panel with expertise in cold plasma and its use in fruit and vegetables to assess the compiled data. Ideally, these data would demonstrate the cold plasma treatment, let us assume with room air, does not leave any chemical residues different from those found on the control strawberries, and the cold plasma treatment does not change the nutritional composition, or other quality factors such as increased retention of strawberry color. If these data are statistical equivalent, then it is likely the GRAS Self-Affirmation panel will conclude that the cold plasma treatment process as prescribed for strawberries does not introduce any food safety concern and would be “Generally Recognized as Safe”. A GRAS Self-Affirmation letter from an expert panel allows one safely to produce a product for sale. FDA is not required to be notified of a GRAS Self-Affirmation determination; however, it is a matter of practice to share the GRAS Self-Affirmation letter with FDA for their information. If they have any regulatory concerns or questions they will generally respond with these queries. The GRAS Self-Affirmation process for regulatory approval of new technology is recommended over the filing of a (indirect) food additive petition when there are no expected residuals. The reason is the FDA regulatory review process has no time limits, and FDA approval for a food additive petition can be over five years, without certainty of approval, since the final signature is from a political appointee overseeing the FDA scientist and their findings. Additionally, if there are concerns, based on the collected data that a residual may exist, then the defined cold plasma treatment process, could add a wash step to remove any excess residuals to ensure the finished product post-treatment remains equivalent to the control product. GRAS Self-Affirmation assessments generally can be completed in one year assuming favorable outcomes in the collected data, indicating no product changes or chemical residuals. More details can be found on the respective US Government websites detailing respective new technology evaluations and making product claims. Additionally, the regulatory approval requirements frequently change as Congress authorizes and regulatory agencies implement additional food safety rules and regulations.

In general, regardless of which agency is the designated technology evaluator, the effect of ACP treatment on the food product needs to be shown as effective, safe, and reproducible. If the ACP treatment leads to a measurable change, such as nutrition, performance, appearance, or otherwise distinguishes it from the untreated (traditional) food article. In that case, additional testing and data must be collected. For those ACP treatments that might create a measurable change of health significance (e.g., increased nitrate levels), possible limits may be established on the treatment process, and final food article testing may need to be performed. In these instances, a consultation with the respective government agencies (FDA, EPA, and/or USDA) will likely be needed. This will require filing the appropriate request for regulatory approval forms and then waiting to secure the necessary meetings. Be forewarned that these conversations can be quite contradictory between each agency as each requires different data to meet their regulatory obligation for approval. In all instances, it is recommended that one seek outside consulting with experience in the regulatory approval process specific to the agency(ies) being engaged.

An important note: data collection for regulatory review and approval needs to be completed on the industrial equipment and not laboratory versions. Thus, significant capital investment is required to build the ACP equipment and generate regulatory data.

One final consideration is the public acceptance of atmospheric cold plasma. ACP will likely be used on the raw agricultural products in many applications and would not require labeling if no significant food article alteration occurs. For those products where the ACP is used on a finished product, the question of “labeling” and “marketing” to the consumer remains an unanswered question.

## 5. Conclusions

The agriculture and food processing sectors are responsible for feeding nutritious and safe food around the world. However, they are also responsible for making the best use of materials and reducing food waste. All these aspects are part of the circular economy concept, where the goal is to reduce waste and take advantage of each part of a product. Processing technologies play an essential role in this concept as it is required to use environmentally friendly tools that do not generate waste or reduce the use of chemicals that cause contamination. ACP technology is becoming the next non-thermal technology in tune with sustainable production for the agriculture and food sector. ACP technology has been widely studied as a decontamination tool for food pathogens, including bacteria, spores, or toxins. The main characteristics are that microbial inactivation can be performed at room temperature, free from food additives, and with an energy-efficient process. This is an attractive technology for thermal-sensitive foods such as fruits and vegetables, deli meats, or fresh food. The interaction of reactive plasma species with the food surface is a complex process that involves mass and energy transfer and other phenomena that still require more development. In addition to its decontamination capacity, the high reactivity of ACP species can be used as a method for the extraction of nutrients, better utilization of materials, or as a technology that allows transforming waste into value.

## Figures and Tables

**Table 1:** Effect of ACP treatment on decontamination, reduction of toxins and pesticides in food.

Target	Product	Description/Results	Equipment	Processing Conditions	Quality	References
***Salmonella* spp.**	Eggs	Spot inoculation, initial load 8.6 log CFU/cm^2^, reduction 4 log CFU/cm^2^@40 s	Arc plasma (APPL-10k Taiwan)	Air, RH * 65%, 12 V/9 A/24,000 W, T < 50C, 0–40 s	Texture, color, pH, acid value, T-bars, fatty acid profile. Not affected	[8]
	Wheat	Mist inoculation, reduction of 4.4 log CFU/g@20 min	DBD plasma, variac/step-up transformer	44 kV, 56.6 W, 60 Hz, air, 0–20 min	NA *	[9]
	Meat	Reduction of 4.7 log CFU/cm^2^, with cold plasma and 200 ppm of peracetic acid	Pulsed DBD plasma (PG 100-3D, Advanced Plasma Solutions, Malvern, PA, USA)	0–30 kV, 0–2 mA, 3.5 kHz, 0–6 min	Color, moisture content. Affected	[10]
***E. coli***	Beef jerky	Combined clove oil and ACP treatment reduced 0.9 log CFU/g@15 min, and 7.5 log CFU/mL on media.	Encapsulated DBD plasma	2.2 kHz, 8.4 kV, 0–15 min	NA	[11]
	Spinach	Reduction of 3.77 log CFU/sample, after storage for 14 days/5 °C	DBD (Phenix Technologies Inc., Accident, MD, USA)	90 kV, 60 Hz, 85% RH, nitrogen gas, 0–5 min	Texture, moisture, color. Not affected	[12]
	Coconut water	Reduction of 5 log@2 min, combined ACP and ascorbic acid	DBD Plasma	90 kV, 60 Hz, 65% O_2–_30% CO_2–_5%N_2_, 0–2 min	Reduced pH, color. Total soluble solids and acidity, not affected.	[13]
***L. monocytogenes***	Radicchio	Reduction 2.2 log CFU/cm^2^@30 min, after 3 days of storage at 4 °C	DBD Plasma	15 kV, 12.5 KHz, 60% RH, air speed 1.5m/s, 0–30 min	Antioxidant activity not affected. Color and sensory analysis.	[14]
**Aflatoxin**	Peanuts	Reduction 38%, increase temperature to 78C@2 min	Plasma jet surface treatment	4.4 kV, 70–90 kHz, 650 W, air flow 107 L/min, 0–2 min	Peroxide value, free fatty acid content, acidity value, oxidative stability index.	[15]
	Milk	Reduction 78.9% Aflatoxin M1@20 min	DBD plasma BK-130 (Phenix Technologies, Accident, MD, USA)	80 kV, 60 Hz, 200 w. Gas: 65% O_2–_30% CO_2–_5%N_2_, 18–22 °C, 0–5 min	Color not affected. pH affected.	[16]
**Fumonisin**	Maize	Reduction 64%@10 min	Plasma jet	6 kV, 20 kHz, mixture of oxygen and 0.75% helium gas, 0–10 min	NA	[17]
**Ochratoxin**	Date palm	Reduction of 24.83 ug/100 mm^2^ with@7.5 min	Plasma jet	25 kV, 25 kHz, 0–9 min	NA	[18]
**Malathion** **Chlorpyrifos**	Lettuce	Degradation 64.6% of malathion, 62.7% chlorpyrifos@180 s	DBD (Phenix Technologies, Accident, MD, USA)	80 kV, 50 Hz, air, 0–180 s	Color and chlorophyll content not affected. Ascorbic acid content.	[19]
**Benazoxystrobin**	Water	Degradation 90%@60 V/9 min	DBD, power supply (CTP-2000 K), axial flow reactor	8.8 kHz, 30–60 V, oxygen gas, 0–9 min	NA	[20]

* RH relative humidity, NA not applicable.
